# Computational prediction of CRISPR cassettes in gut metagenome samples from Chinese type-2 diabetic patients and healthy controls

**DOI:** 10.1186/s12918-015-0248-x

**Published:** 2016-01-11

**Authors:** Tatiana C. Mangericao, Zhanhao Peng, Xuegong Zhang

**Affiliations:** MOE Key Lab of Bioinformatics/Bioinformatics Division, TNLIST/Center for Synthetic and Systems Biology, and Department of Automation, Tsinghua University, Beijing, 100084 China; Department of Bioengineering, Instituto Superior Técnico (IST), Lisbon, Portugal; School of Life Sciences, Tsinghua University, Beijing, 100084 China

**Keywords:** CRISPR, Human gut microbiome, Metagenome

## Abstract

**Background:**

CRISPR has been becoming a hot topic as a powerful technique for genome editing for human and other higher organisms. The original CRISPR-Cas (Clustered Regularly Interspaced Short Palindromic Repeats coupled with CRISPR-associated proteins) is an important adaptive defence system for prokaryotes that provides resistance against invading elements such as viruses and plasmids. A CRISPR cassette contains short nucleotide sequences called **s**pacers. These unique regions retain a history of the interactions between prokaryotes and their invaders in individual strains and ecosystems. One important ecosystem in the human body is the human gut, a rich habitat populated by a great diversity of microorganisms. Gut microbiomes are important for human physiology and health. Metagenome sequencing has been widely applied for studying the gut microbiomes. Most efforts in metagenome study has been focused on profiling taxa compositions and gene catalogues and identifying their associations with human health. Less attention has been paid to the analysis of the ecosystems of microbiomes themselves especially their CRISPR composition.

**Results:**

We conducted a preliminary analysis of CRISPR sequences in a human gut metagenomic data set of Chinese individuals of type-2 diabetes patients and healthy controls. Applying an available CRISPR-identification algorithm, PILER-CR, we identified 3169 CRISPR cassettes in the data, from which we constructed a set of 1302 unique repeat sequences and 36,709 spacers. A more extensive analysis was made for the CRISPR repeats: these repeats were submitted to a more comprehensive clustering and classification using the web server tool CRISPRmap. All repeats were compared with known CRISPRs in the database CRISPRdb. A total of 784 repeats had matches in the database, and the remaining 518 repeats from our set are potentially novel ones.

**Conclusions:**

The computational analysis of CRISPR composition based contigs of metagenome sequencing data is feasible. It provides an efficient approach for finding potential novel CRISPR arrays and for analysing the ecosystem and history of human microbiomes.

**Electronic supplementary material:**

The online version of this article (doi:10.1186/s12918-015-0248-x) contains supplementary material, which is available to authorized users.

## Background

The human body is host to this complex community of symbiotic, pathogenic and commensal microorganisms (microbiome), whose abundance is estimated to exceed the number of human cells by at least an order of magnitude [1]. It has been estimated that the microbes in our bodies collectively make up to 100 trillion cells, tenfold the number of human cells [2]. To understand and exploit the impact of the microbes on human health and wellbeing, it is necessary to interpret the content, diversity and function of the microbial community. Metagenomic sequencing has been proven to be a powerful tool for analysing complex microbial communities. And in this context many projects have been developed all over the world to study human microbiomes of multiple body sites. For example, the US-based Human Microbiome Project (HMP) [3] and the EU-based MetaHIT project [4] have generated resources that can enable the comprehensive characterization of the human microbiome and analysis of its role in human health and disease.

Among all body sites, the diversity of microorganisms in the human gut is known to be among the highest [1]. This community has been discovered to be associated with human physiology through processes related to development, nutrition, immunity, and resistance to pathogens [5–9]. While bacteria are responsible for these functions and most research has focused on the interaction of bacteria with the host, bacteriophages, in turn, influence the composition and abundances of bacteria in the human gut [10, 11].

Bacteria and Archaea have evolved defence and regulatory mechanisms to cope with various environmental stressors especially virus attacks. The understanding on this arsenal has been expanded by the discovery of the versatile CRISPR-*Cas* system. Bacteria can remember their viral invaders by sampling short DNA sequences, known as protospacers, from the genetic materials of viruses or phage. These sequences become integrated into the bacterium’s own DNA, specifically into an array of repeat sequences called clustered regularly interspaced short palindromic repeats (CRISPR). The integrated sequences are called spacers [12]. When these sequences are transcribed and processed into small RNAs, they guide a multifunctional protein complex (*Cas* proteins – CRISPR associated proteins) to recognize and cleave incoming foreign genetic material [13]. The diversity of *Cas* genes suggests that multiple pathways have been developed to use the basic information contained in the CRISPR cassettes in diverse defence mechanisms [14]. This adaptive immunity system was first observed in *Escherichia coli* in 1987, although its significance was not straightaway apparent. Since then, CRISPR arrays have been identified in approximately 40 % of Bacteria and 90 % of Archaea [15].

CRISPR cassettes were already characterized across human body sites in different individuals in independent projects [16–18] and as a part of the Human Microbiome Project (HMP) [14] with particular focus in the gut metagenome [14, 19, 20]. So far there have been two main approaches for the study of CRISPR in metagenomic samples: one focuses more on the analysis of spacers in raw reads, which are then used to search for CRISPR cassettes [19]; the other is based on the reconstruction of CRISPR arrays, where direct repeat consensus sequences from known CRISPR types identified in reference genomes are used to recruit reads from the dataset and then be assembled into CRISPR loci [14]. For the present work, and since we didn’t have access to raw reads, we decided to follow a third approach, which is based on the identification of CRISPR cassettes in assembled contigs/scaffolds [21]. This approach differentiates from the previous ones because it allows finding novel CRISPR cassettes, as *de novo* prediction of CRISPRs relies on sequence features of CRISPRs that don’t exist in short reads. Partially following the strategy of Gogleva et al [21], we analysed the CRISPR composition of the metagenomic data of a set of Chinese individuals [22]. The findings were compared with those on the 3 different datasets used in Gogleva et al [21].

## Materials and methods

### Metagenomic datasets

We used the gut metagenome data of a set of Chinese individuals published in [22]. The data contain 145 individuals, with their gut microbiota sequenced using whole genome sequencing [22]. The samples include 71 type-2 diabetic individuals and 74 non-diabetic individuals used as controls. Individual metagenomes were assembled in contigs with the average size of 10,687 bp. The the total length of contigs comprised 15.96 Gb. More information about the samples are provided in Additional file 1.

### Identification and analysis of CRISPR cassettes

To construct a set of CRISPR cassettes from the metagenomic dataset, we used the software PILER-CR [23]. PILER-CR searches for CRISPR repeats by doing a multiple alignment between them in each array. It also shows flanking regions and spacers, making it visually obvious when PILER-CR mis-identifies the repeat boundary by one or a few positions. In the output, repeats are clustered by similarity to reveal relationships between different arrays and, where applicable, to provide confirmatory evidence of marginal arrays (i.e. arrays with only 3 or 4 repeats that may not be perfectly conserved). Sequences are considered similar, i.e., clustered in similar groups, if they have an estimated mutual identity of more than 50 %. The parameters used in this work followed the default parameters proposed by PILER-CR, (Table 1). With PILER-CR it is possible to extract the repeat consensus sequences and the spacers’ sequences. There is information about the array size, as well as information about where the CRISPR array is located (which scaffold/contig).

**Table 1:** PILER-CR algorithm parameters

Parameter	Value	Description
Minrepeat	16	Minimum length of a repeat.
Maxrepeat	64	Maximum length of a repeat.
Minspacer	8	Minimum spacer length.
Minarray	3	Minimum number of repeats in an array/cassette.
Maxspacer	64	Maximum spacer length.
Minrepeat ratio	0,9	Repeat length ratio
Minspacer ratio	0,75	Spacer length ratio
Mincons	0,9	A run of minarray repeats with at least mincons similarity is required for an array to be reported. The value is specified as fractional identity 0.0… 1.0, with 1.0 meaning identical sequences.

### Repeat clustering and the CRISPRmap tool

Repeat consensus sequences were submitted to the standard BLASTClust procedure to form repeat clusters (parameters: -L 0.5 -S 50 -e F -p F -W 15; L is length coverage threshold, S is bit score coverage, default is 1.75) [24]. BLASTClust clusters nucleotide sequences based on pairwise matches found using the MegaBLAST algorithm for DNA/nucleotides. A single MegaBLAST search is performed for all the sequences combined against a database created from the same sequences. BLASTClust finds pairs of sequences that have statistically significant matches and clusters them using single-linkage clustering [24]. We used a first BLASTClust procedure as a filtering step to find identical repeats and construct a set of unique repeat sequences. And a second BLASTClust procedure was used to form clusters between unique repeat sequences previously found.

Another tool used for repeat clustering and analysis was CRISPRmap [25]. This tool allows CRISPR repeats to be divided into types as part of a comprehensive independent clustering analysis to determine conserved sequence families, potential structure motifs for endoribonucleases, and evolutionary relationships [25]. The repeats are divided among 6 superclasses, named A to F, that comprise 24 families and 18 structural motifs.

### Repeat BLAST to CRISPR database

Unique repeat consensus sequences were matched against the CRISPR database CRISPRdb [26] (http://crispr.u-psud.fr/crispr/BLAST/CRISPRsBlast.php) using a standard BLAST with an *e-value* threshold of 0.01, in order to find hits for known repeats in our findings and to access the possibility of new repeat sequences. We added a step to remove the matches that have continuous indels of more than 5 nucleotides at either ends of the query or target sequences.

## Results and discussions

### Characteristics of CRISPR

We used data from the Chinese type-2 diabetes gut microbiota study [22] to search for CRISPR cassettes in gut metagenome sequences using PILER-CR with a similar pipeline as in Gogleva et al [21]. In the original pipeline by Gogleva et. al. [21], they also applied both the software CRT (CRISPR Recognition Tool) [27] and CRISPR Finder [28] to the dataset in order to find CRISPR cassettes. However, there are some issues in using these software: CRISPR Finder is an online tool and it’s not available for download. Trying to upload large data to the website makes the processing time too long; for CRT, on the other hand, although it is available for offline usage, it also has some usage problems since it considers scaffolds of each individual as connected to each other as a single sequence, which is incorrect and can cause unreliable results. In further research, we should find a way for the program to consider each scaffold individually and make CRT results comparable to PILER-CR results. But for the current project, we chose only to use PILER-CR for this step.

Applying PILER-CR with default parameters, we found a total of 3169 candidate CRISPR cassettes. Given the said conditions, a filtering system was not applied, and all the CRISPR cassettes found were kept for further analysis. Among the total number of CRISPRs found, 1624 cassettes belong to the 74 individuals from the control group and the remaining 1545 cassettes belong to the 71 individuals with type-2 diabetes. CRISPR cassettes have been predicted in all single individuals. The full set of identified cassettes is shown and characterized in Additional file 2.

The identified 3169 CRISPR cassettes comprised a total of 36,709 spacers and 1302 unique repeats, meaning that only about 40 % of the repeats were unique. This could imply that the same CRISPR-containing bacteria were recurrent in multiple individuals. Within this dataset, there is an average of 22 CRISPR array per individual, and each cassette contains an average of 12 spacers, hence the average size of 767 bp per CRISPR. It should be referred that if we analyse both healthy and diabetic individuals separately the results are very similar, meaning that we couldn’t find an obvious distinction between the number of CRISPR cassettes or number of spacers found (Table 2).

**Table 2:** Summary of the results’ comparison between the set of CRISPR cassetes found for type-2 diabetic individuals and healthy individuals

	Metagenomic dataset group	Type-2 diabetic individuals	Healthy (non-diabetic) individuals
	Cassettes		
Identified by PILER-CR		1624	1545
Average Length (in bp)		757	777
	Spacers		
Total number		16 949	17 547
	Repeats		
Average size (in bp)		31	32

Comparing our findings with the ones from Gogleva et al. [21] on gut samples of from 124 European individuals, downloaded as an assembly of 1,889,651 contigs with a total size of 3732 Mb (http://public.genomics.org.cn/BGI/gutmeta/UniSet/), the most similar dataset to the one we used in terms of data size, we found that the number of CRISPR cassettes predicted by PILER-CR in our work was larger. They initially predicted 121 CRISPR cassettes for 124 individuals. After the filtering process, their reliable set of CRISPR had a total of 78 cassettes including 378 spacers and 74 unique repeats. According to Gogleva et al.’s results from the 3 software they used, PILER-CR presented the least number of predicted CRISPR cassettes, which may be explained by its high specificity considering genomic repeats and low-complexity regions as a candidate CRISPR cassette, a process followed by several refinement steps [21, 23].

The relatively large number of predicted CRISPR cassettes in our work was not filtered with extra criteria. However, based on the results of filtering in the reference, we can predict that, even if we applied those filtering steps, the number of reliable CRISPR cassettes in our data would still be larger than that in Gogleva et al. [21]. This might lead us to suggest that the gut microbiota of the Chinese individuals, compared with European individuals, is composed by a larger number of bacteria “armed” with CRISPR adaptive defence mechanism. But the current data are insufficient to support such an observation. A more possible reason that might cause the difference in the number of identified CRISPR cassettes is that the two metagenome data were sequenced at least two years apart in time, which is a period when sequencing technology and protocols have developed very rapidly. Differences in sequencing protocol settings in the data may cause differences in predicting CRISPRs from the data. The data we used has 8,039,994 contigs with the total length of 16,345 Mb, which are much larger than the data used in Gogleva et al.. Besides data size, the number of CRISPRs found is conditioned by a series of other known or unknown factors such as types of bacteria present in the microbiota of the individual, the size of CRISPR systems which can dependent on the history of horizontal gene transfer and former phage invasions, the program used to search for CRISPR, protocols and settings in the sequencing experiment, and the quality of the sequencing data and assembly of the metagenomic sample, etc.. The observations in this study suggested that more studies on both real and simulated data are needed to build a better understanding of the distribution of CRISPRs in metagenome data and to develop better methods/strategies for predicting CRISPRs in metagenome data. The summary of the comparison of the results is given in Table 3.

**Table 3:** Summary of the results’ comparison between the CRISPR analysis in this present work and Gogleva et al

	Study	In Gogleva et al., 2014	The present work
	Cassettes		
Identified by PILER-CR		121	3169
Average length		338 bp	767 bp
	Spacers		
Total number		378	36 709
	Repeats		
Total number		121	3169
Unique repeats		74	1305

### Repeat clustering with CRISPRmap

After collecting the CRISPR cassettes that resulted from the PILER-CR software, we applied a standard BLASTClust procedure and also submitted our data to the CRISPRmap web server. BLASTClust was used in the first phase to find and construct a set of unique repeat sequences. In this work, an ID number or the denomination of “RepeatCluster” was attributed to the repeats. The latter refers to the repeat sequences that are identical and shared by more than two CRISPR arrays. As stated above, the set of unique repeats was composed by a total of 1302 repeat sequences. The set of unique repeats is available in Additional file 3A.

In the second phase, we constructed repeat clusters based on sequence similarity. See Additional file 3B. We consider groups of two or more repeats as a cluster. In this way 121 repeat clusters were formed. The majority of the clusters (68 out of 121) are composed by two repeats. Another 37 clusters contained three or four repeats. The biggest cluster contained 12 repeats. Since BLASTClust can only cluster sequences of high similarity (few mismatches) we consider it can be used as a reliable tool to find identical repeats from an “ocean” of sequences.

CRISPRmap was designed for independent and comprehensive classification of all known CRISPR cassettes based entirely on the repeat properties [25]. Their approach considers not only the pairwise similarities between repeats, but also how the binding affinity of *Cas* proteins is affected by the repeat structure, namely, a small hairpin structure (a key binding motif for *Cas* endoribonucleases in several systems) [25]. Their classification system provides a more complete insight into the diversity of CRISPR systems. Applying CRISPRmap to our identified CRISPR repeats we found that 670 repeats were attributed superclasses, while the remaining 632 repeats were not assigned to any superclass. Additional file 4 provides the CRISPRmap results. Representatives for all superclasses were found. The most populated superclasses are: superclass A with 138 unique repeats, superclass C with 168 unique repeats, and superclass F with 182 unique repeats. To 365 out of 1302 repeats it was also attributed a structure motif. It’s observed that for the majority of repeats belonging to the same superclass, the structure motif is similar. The summary on the superclasses is given in Table 4.

**Table 4:** CRISPR map clustering of the collection of unique repeat sequences from Chinese individuals’ CRISPR cassettes

	Number of repeats	Sequence families associated	Motifs
Superclass A	138	2, 7	6
Superclass B	102	1,6	1,2,7
Superclass C	168	5	3,4
Superclass D	32	None	11
Superclass E	48	9,24	3,4,15,16
Superclass F	182	13,23	3,4,15,17,18

The superclasses were ordered according to generally decreasing conservation. Superclass A contains highly conserved CRISPRs repeats at the sequence level, but only a few small structure motifs. Superclasses B and C contain sequence families that roughly correspond to one structure motif each; the same is true for half of superclass D. The other half of superclass D and superclass E contain little sequence conservation, but many small conserved motifs. The bacterial repeats in superclass F are divergent [25]. For the repeats to which a superclass was attributed, 78 of those was also attributed to a sequence family. It’s noted that families are associated with superclasses, and to no repeat was assigned a sequence family but not a superclass.

### Repeat BLAST to CRISPRdb database

Our set of unique repeat sequences was then compared to the already known repeats from CRISPR cassettes collected in CRISPRdb [26] (e-value threshold of 0.01). CRISPRdb is a public database which is updated monthly from newly released genome sequences, to display CRISPRs and to generate dictionaries of spacers and repeats. This database is constructed using CRISPRFinder as a CRISPR identifying software. At the moment, they have in their database a total of 2612 bacterial genomes analysed (last updated 2015-08-05) where they found 1176 strains with convincing CRISPR(s) and 126 out of 150 Archae genomes with convincing CRISPR(s).

From our collected set of repeats, 932 out of the 1302 unique repeats had matches in the CRISPRdb. However, 148 of them have continuous indels at either ends of the query or target sequences. They may be overlapping repeats from different CRISPRs. We took them as unmatched repeats to keep higher confidence in the matched repeats. The repeat sequences that weren’t matched to CRISPRdb can possibly be of novel CRISPR cassettes that have not been reported before. The detailed results are given in Additional file 5. In total, a number of 190 different strains were found. The majority of the matches belonged to *Megamonas hypermegale ART12/1*, although the number of matches isn’t particularly relevant since it only ends up representing 3 % of the total matches. The second most matched strains, within our repeats, were *Bacteroides helcogenes P36-108*, *Eubacterium rectale M104/1* and *Faecalibacterium prausnitzii SL3/3*, all strains commonly found in the human gut microbiota [29, 30]. The last two strains are bacteria that are part of the colonic microbiomes responsible for the fermentation of hexose and pentose sugars [31]. The results obtained may not be completely accurate, but they indicated a large diversity of CRISPR-containing strains present in the human gut microbiome.

### Comparison of CRISPR repeats found in different data

We compared the findings from the diabetics patient group and the control group. The diabetics group has 3,777,094 contigs. A total of 1624 repeats were identified, with 790 unique repeats. The control group has 4,262,900 contigs. A total of 1545 repeats were found, with only 600 unique repeats. There are 88 unique repeats shared between the two groups. Additional file 3 shows unique repeats in two groups.

We also compared using BLASTclust the repeats we found with those found by Gogleva et. al. [21] on the three datasets they used: the European data of 124 individuals, the JPN data of 13 healthy Japanese individuals, and the DG data of 2 healthy adults in the Distal Gut metagenomic project. The latter two data were sequenced with different sequencing platforms. In the DG results, 4 out of 14 unique repeats were clustered into the same group with the unique repeats we found in the Chinese dataset. In the results on the European data, 7 out of 74 unique repeats were clustered with those in our data. In the results on the JPN data, 119 out of 296 are clustered with unique repeats we found in the Chinese data, which means 40 % of the repeats are highly similar with the unique repeats we’ve analysed. This may suggest that the two Asian gut metagenome datasets share higher similarity in terms of CRISPR compositions, but further investigation is needed for a systematic comparison.

## Conclusions

We analysed CRISPR content in a human gut metagenomic dataset of Chinese individuals of healthy and type-2 diabetes groups. With some newly released tools for CRISPR identification and post-processing, we were able to profile CRISPR cassettes and their corresponding repeats and spacers in the gut microbiome of this sample set. Comparison with the existing database show that the majority of the identified CRISPRs have been reported in the literature, while there are a quarter of the identifications indicate newly discovered CRISPRs that have not been reported before.

The human gut microbiota is one of the most complicated microbial ecosystems in the human body and has important associations with human health. CRISPR is a major system that microbes use to deal with phage. Therefore analysing the CRISPR composition of a microbiome and of a group of microbiomes can be very informative for understanding the history and function of the microbiome. Also, the microbiota is composed of highly diverse bacteria species that cannot be cultured. Identifying new CRISPRs from metagenome data might also provide an efficient approach for finding possible novel CRISPRs that may be used for genome editing applications.

The collection of spacers and repeats constructed in this work constitutes a base for further studies. The preliminary comparison between CRISPRs found in different groups of data and between different studies showed interesting observations. As multiple gut metagenome datasets have been published in the projects like HMP, MetaHIT and other projects that focus on specific groups of people or specific human diseases, it’ll be interesting and promising to conduct systematic comparative study among different datasets, which may lead to better understanding of the forming, shaping, changing and function of gut microbiome populations in different people.

The current strategy for computational profiling of CRISPRs is still preliminary as there has been no stringent control on the possible false discoveries. Some filtering steps may be necessary to reduce false discoveries. For example, Gogleva et al. [21] suggested three steps to form a reliable set of CRISPR cassettes: (1) Cassettes predicted by more than two CRISPR predicting programs, should be kept. Using more than one algorithm is recommended as they have complementary strengths for precision and recall; (2) Cassettes predicted by less than two programs, i.e. candidate cassettes, which are adjacent to *cas* genes, are added to the set of cassettes constructed in the first step; (3) candidate cassettes whose repeat consensus sequence are part of a repeat cluster containing repeats from already established as reliable CRISPR cassettes, are added to the set. This strategy can be further calibrated and improved when more data and features are available.

More advanced studies could investigate more on the *Cas* proteins and their relationship with CRISPR array repeats, for better understanding of the defence mechanism. Methods like CRISPRmap are important for further studies regarding CRISPR-*Cas* proteins systems. We should also pay more attention to the spacer sequences and, for example, on discovering how this defence mechanism recognizes short sequence motifs, known to be adjacent to the spacer precursor in the invaders genomes.

## Additional files

Additional file 1:
**Information of the metagenome samples.** The Chinese dataset consisted in faecal samples belonging to 145 Chinese individuals living in the south of China, collected by Shenzhen Second People’s Hospital, Peking University Shenzhen Hospital and Medical Research Center of Guangdong General Hospital. The set comprised 71 type-2 diabetic individuals - classified DLF, DLM, DOF and DOM - and 74 healthy controls - NLF, NLM, NOF and NOM. (XLSX 27 kb) Additional file 2:
**PILER-CR predicted CRISPR cassettes.** This file shows the detailed info about the predicted CRISPR cassettes with PILER-CR software. The number of cassettes found in each individual, cassette origin scaffold, length (in base pairs), number of spacers and repeat consensus sequences. (XLSX 257 kb) Additional file 3:
**A: BLASTClust results.** Table S4A includes the collection of unique repeat sequences and Table S4B the results of BLASTClust alignment. (XLSX 60 kb) Additional file 4:
**CRISPRmap results.** The results for CRISPRmap contain the distribution of the unique repeat sequences for the 6 superclasses (A-F), sequence families and structural motifs. There is a summary table of the results as well as a detailed table with data about the sequence families. (XLSX 90 kb) Additional file 5:
**Repeat sequence’s BLAST hits against CRISPRdb database.** The results for the BLAST search against CRISPRdb of known repeats include a table showing for each unique repeat consensus the corresponding hit in the database, strain name and NCBI code, as well as the *e-value*. The excel file also includes a summary of the results (percentage of unique repeats that found a hit in the database), as well as the distribution of the known repeats for the different Bacteria *phylum*. Those removed repeat sequences that have continuous indels at either ends of the hits are shown in Table S5C. (XLSX 147 kb)
